# Person-Specific Gaze Estimation from Low-Quality Webcam Images

**DOI:** 10.3390/s23084138

**Published:** 2023-04-20

**Authors:** Mohd Faizan Ansari, Pawel Kasprowski, Peter Peer

**Affiliations:** 1 Department of Applied Informatics, Silesian University of Technology, 44-100 Gliwice, Poland; 2 Faculty of Computer and Information Science, University of Ljubljana, Večna Pot 113, SI-1000 Ljubljana, Slovenia

**Keywords:** gaze estimation, convolution neural network, computer vision, deep learning

## Abstract

Gaze estimation is an established research problem in computer vision. It has various applications in real life, from human–computer interactions to health care and virtual reality, making it more viable for the research community. Due to the significant success of deep learning techniques in other computer vision tasks—for example, image classification, object detection, object segmentation, and object tracking—deep learning-based gaze estimation has also received more attention in recent years. This paper uses a convolutional neural network (CNN) for person-specific gaze estimation. The person-specific gaze estimation utilizes a single model trained for one individual user, contrary to the commonly-used generalized models trained on multiple people’s data. We utilized only low-quality images directly collected from a standard desktop webcam, so our method can be applied to any computer system equipped with such a camera without additional hardware requirements. First, we used the web camera to collect a dataset of face and eye images. Then, we tested different combinations of CNN parameters, including the learning and dropout rates. Our findings show that building a person-specific eye-tracking model produces better results with a selection of good hyperparameters when compared to universal models that are trained on multiple users’ data. In particular, we achieved the best results for the left eye with 38.20 MAE (Mean Absolute Error) in pixels, the right eye with 36.01 MAE, both eyes combined with 51.18 MAE, and the whole face with 30.09 MAE, which is equivalent to approximately 1.45 degrees for the left eye, 1.37 degrees for the right eye, 1.98 degrees for both eyes combined, and 1.14 degrees for full-face images.

## 1. Introduction

Gaze estimation is a significant research problem due to its various applications in real life, including human–computer interactions [1], behavioral analysis [2], virtual reality [3,4], and health care [5]. Since a person’s gaze is an observable indicator of visual attention, eye movement research started back in the early 19th century [6]. Real-time eye-tracking was possible back in the 1980s; however, at that time, eye-tracking applications were limited to cognitive and psychological processes and medical research. Nowadays, due to immense improvements in computing speed, low-cost hardware, and digital video processing, gaze tracking tools are becoming available for new applications, such as virtual reality, web advertisements, and gaming [7].

At present, there are many commercial eye trackers available. The typical desktop eye tracker’s accuracy is between 1 and 2 degrees, and the best commercial eye tracker’s accuracy is about 0.5 degrees [8,9]. Furthermore, there are many solutions available for gaze estimation. However, most of them suffer from high cost [10], invasive hardware [10], or imperfections under real-world conditions [11,12,13]. All these factors are the main reason that prevents eye-tracking technology from being available to everyone with just a desktop computer, laptop, or smartphone. Our ultimate goal is to address these limitations and make eye-tracking available to everyone, even with limited resources.

We believe we can achieve this milestone by building a system that can work on desktop or mobile devices without the need for other instruments with an accuracy comparable to commercial devices. We considered desktop or laptop devices for this particular task since, compared to a laptop, mobile devices currently have excellent built-in cameras that can produce high-quality images. However, our goal is to build an eye-tracking algorithm that can work on low-quality images such as those from laptops or desktop devices that have standard cameras.

The recent success of deep learning techniques has revolutionized different domains, including computer vision tasks such as image recognition [14], object detection [15], object tracking [16], and segmentation [17]. However, success is limited in eye tracking due to lack of data availability [12,13,18]. In this work, we attempted gaze estimation for a specific person and collected our own dataset using the desktop application. We tested different combinations of the left eye, right eye, and face regions for gaze estimation. Furthermore, we tested different CNN architectures and tuned their parameters to get the optimal model. However, we do not explicitly claim that our CNN network is better than other networks used for the same purpose.

The main contributions of this paper are as follows:

- We collected a dataset of low-quality eye and face images that may be used to train deep CNN models.
- We analyzed different hyperparameters of the CNN network, showing that their values have a significant impact on final results.
- We showed that the model created for one person produces far fewer errors than the general models trained on multiple users’ data and that this error rate is comparable to commercial eye trackers with a value below 2 degrees of visual angle.

The rest of the paper is structured as follows: Section 2 presents the previous work related to gaze estimation. In Section 3, we provide information about the dataset, different combinations of CNN parameters, and the CNN architecture used. Section 4 presents the results achieved in this study. Finally, Section 5 and Section 6 present the discussion, conclusion, and our future work related to gaze estimation.

## 2. Related Work

Due to the wide range of applications in various domains, gaze estimation has been studied for decades. Video-based oculography (VOG) is the most popular method nowadays. Generally, VOG-based gaze estimation methods can be divided into two categories: model-based, also known as feature-based, and appearance-based methods [19]. Model-based methods rely on hand-crafted features of eye regions like limbus, pupil, and corneal reflection for gaze direction [20]. Furthermore, these methods also require an infrared light source and high-quality cameras to capture eye regions with good quality. Model-based methods can accurately predict gaze coordinates, but they require specialized hardware, which limits their applications [20].

Appearance-based methods learn a gaze direction directly from the images, either from the eyes [13,21,22,23,24] or from the face [10,25]. There are many datasets available for gaze estimation [10,11,12,13,18,23,25,26,27,28,29,30]. However, learning gaze directly from images is challenging because of changes in head movement, different light conditions, eye positions, and occlusion [31]. Therefore, appearance-based gaze methods require large amounts of diverse training data. Appearance-based methods can be trained with user-specific data [24]. However, due to the practical limitation of collecting a large amount of user-specific data, multiple user approaches get more attention for gaze estimation [24]. In this paper, we try to build a personalized model using user-specific data collected from a webcam.

Early work in appearance-based gaze estimation was restricted to the laboratory environment with a fixed head pose because of its simplicity [32,33,34]. Moving forward with more development, this restriction started to relax with the introduction of new datasets collected in unconstrained environments [10,13] or synthetic datasets [12,35,36]. The availability of large-scale datasets has given researchers the opportunity to conduct experiments using a wide variety of methods, including different regression methods [11,37], random forest [12], k-nearest neighbors [36], and a variety of CNN architectures [10,13,35,38,39]. Among all methods, CNN methods have been shown to be the most robust for gaze estimation when given a sufficient amount of data for training the CNN network.

Many CNN architectures have been proposed by researchers for gaze estimation, primarily for person-independent gaze estimation. For instance, in [10], the authors proposed a GazeCapture dataset and then trained a CNN on this dataset. Their network takes the face and eye images with their location associated with the face grid for gaze estimation. Authors of [25] proposed a gaze estimation method using face images only for 2D and 3D settings. They proposed a special weight mechanism that takes the information from different parts of the face and learns the gaze coordinates using a standard CNN network. Furthermore, in [40], authors proposed a framework for person-specific gaze behavior with a small number of calibration samples. Their method learns a rotation-aware latent representation of gaze with a disentangled encoder–decoder network that uses meta-learning to train the gaze estimator. Recently, authors of [41] proposed a multi-person real-time gaze estimation. They proposed a first one-stage gaze estimation method (GazeOnce) that estimates human gaze direction in one pass. Furthermore, they also offered a new dataset MPSGaze for multi-person gaze estimation. In another paper [42], authors proposed MTGLS: a novel multitask gaze estimation framework that requires limited supervision for rich visual representation learning. They demonstrated the effectiveness of their proposed method on CAVE, MPIIGaze, Gaze360, and DGW datasets.

## 3. Materials and Methods

This section provides information about data collection for the experiment, along with a description of the necessary data preprocessing required for deep neural networks. Further, we provide information about the dataset and the overview of the network architectures used in this study.

### 3.1. Data Collection

This section presents information about the data collection procedure, which plays an essential role in deep learning techniques. The quality of data on which the network is trained has a significant impact on the overall performance of the network.

**Figure 1:**
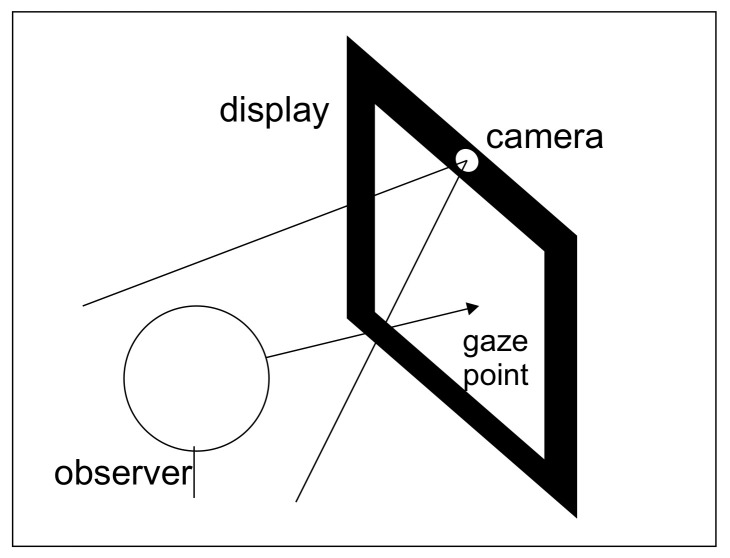
The experimental setup [43].

The data used for the experiment were collected using a DataCollector desktop application which collects images from a built-in laptop camera. We used the MSI GF75 Thin Core i7 9th Gen laptop in this research. The laptop camera resolution was 640 × 480 pixels during the data collection. No other hardware was required for this data collection procedure except a webcam, preferably on the top of the desktop screen as in Figure 1 [43]. This simple procedure allowed us to collect data under real-world scenarios, rather than in a strict laboratory environment. The test subject was asked to look at the different points on the screen while seated from 30 to 35 cm from the screen. The test subject was also asked to pay attention to the lighting conditions during the data collection procedure because different lighting conditions heavily affect the quality of the image. Failure to pay attention to the lighting conditions sometimes resulted in very low-quality, totally dark, or half-dark images (sometimes one eye was visible while the other was not). The data were collected in 22 sessions over several days, at various locations, and at different times of the day and night. Every session lasted from one to two minutes. This approach aimed to ensure that the dataset was diverse and included variability. The image quality was better during the day when the lighting conditions were ideal, while the image quality was low during the night due to the low light in the room and surrounding environment. Therefore, the dataset includes images collected in different lighting conditions that provide an opportunity to test the model in diverse settings. The variability in the dataset also enhances the robustness of the models trained on it, which allows for more generalized results. During each data collection session, the subject was asked to click on 54 points on the screen. The points were randomly selected, i.e., the user could click any point but was encouraged to maintain equal distribution of points on the whole screen. With each click, ten images of the person’s face were collected and stored along with the person’s gaze location. The dataset created for this research is distinct from other universal data (collected from multiple users to train the model), as it was specifically collected for an individual user.

### 3.2. Data Preprocessing

**Figure 2:**
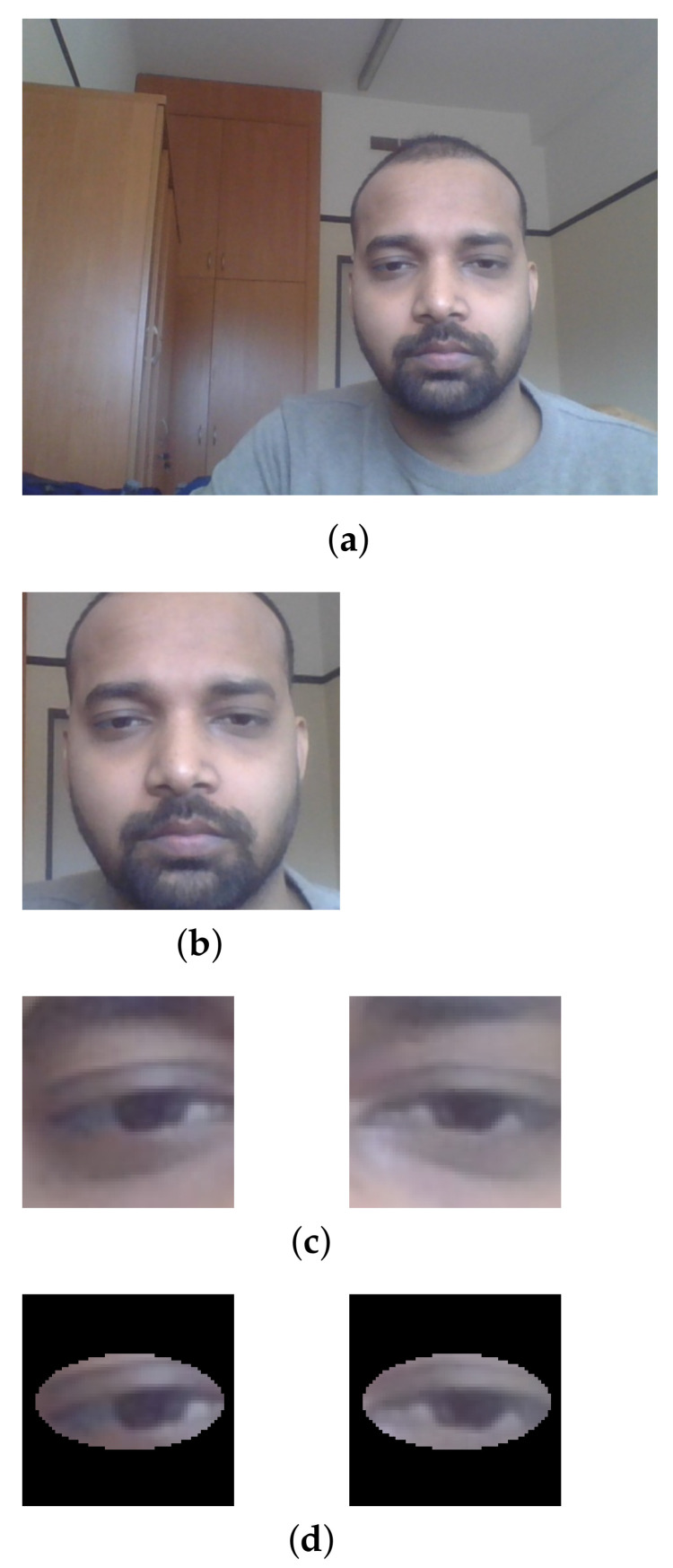
Pre-processing steps. (**a**) Step 1: Original image. (**b**) Step 2: Face detection. (**c**) Step 3: Eye detection. (**d**) Step 4: Eye masking.

After data collection, the next step was to perform data preprocessing, which is an important step in any machine learning or deep learning technique. Figure 2 presents all preprocessing steps. The first step in Figure 2a was to take an image from the webcam, and the next step in Figure 2b was to detect face using the Viola–Jones (VJ) classifier that uses the Haar cascade [44]. We used VJ because it is easy to use. All incorrectly-classified images were manually removed. In the next step, the left eye and right eye (Figure 2c) were detected using another Haar cascade. This approach reduced the chance of misclassifying eyes in images by detecting objects that are similar to eyes [43]. The average dimensions for the left eye image were 55.12 × 55.12 pixels with standard deviation 8.76; for the right eye 55.89 × 55.89 pixels with standard deviation 7.23; and for the face image 223.24 × 223.24 pixels with standard deviation 23.29. Before sending data to the CNN, all eye images were resized to 64 × 64 pixels and face images to 224 × 224 pixels.

The data collected with this approach had to be checked manually and verified before feeding the neural network. It includes checking all images to see if they contain accurate information. There were situations when the Haar cascade for eyes detected similar objects resembling an eye. We manually checked such images and removed them from the dataset. Furthermore, sometimes, a person blinked or closed their eyes during recording. Such samples should not be included in the final training. We manually removed such images from the dataset before training.

The dataset was collected for person-specific gaze estimation. A total of 11,800 images were collected for the experiment. Further, we also made another dataset by masking the eyes’ surroundings with a white ellipse with a black border (Figure 2d). We included this procedure to check the effect of minimizing the influence of neighboring pixels. The dataset was split in an 80/20 manner for training and testing of different models.

### 3.3. Convolutional Neural Network Architecture

This section presents information about different architectures used in the study. We used the CNN network architecture because it is the state-of-the-art solution for image analysis. We checked two architectures: with input as one image and input as two images. For one-image input, we used images of only the left eye, only the right eye, both as is and masked, and full-face images. For two-image input, we combined left and right eye images.

The network output was a vector of two values representing the location of a person’s eye gaze on the screen. Every tested network consisted of five layer types:

1. Convolutional layers that learn the feature map from the input image.
2. Pooling layers that reduce the image.
3. Batch normalization layers used to stabilize the neural network.
4. Dropout layers used to prevent over-fitting during training.
5. Fully-connected layers that calculate the final output.

The ADAM optimizer [45] was used to train the network with a different set of learning rates while tuning the network parameters. After finding the optimal value for the learning rate, we re-trained the network with this value. We used the rectified linear unit (ReLu) [46] as an activation function in the convolutional and fully-connected layers. Default weight initialization for filters was used from the Keras implementation. The MAE function was used as a loss function during training. Equation (1) represents the mathematical formula for MAE:

(1) $$\mathrm{MAE}=\frac{\sum_{i=1}^{N}\left|y-\bar{y}\right|}{N},$$

where *y* is the actual value, $\bar{y}$ is the value predicted by the network, and *N* is the total number of examples.

An identical naming convention was adopted for a single eye, double eye, face, single eye with ellipse (mask), and both eyes with ellipse experiments. The LE symbol represents only the left eye as input; RE represents only the right eye; FF represents the full face as input; BE represents both left and right eyes; LEM represents the left eye with ellipse (LeftEyeMasked); REM represents the right eye with ellipse (RightEyeMasked); and BEM represents both left and right eyes with mask (BothEyesMasked).

The CNN architecture for a single eye and face consists of 3 convolutional layers, 1 pooling layer, 2 batch normalization layers, 1 fully-connected layer also known as a dense layer, 1 dropout layer, and 1 output layer, which is also a dense layer but with 2 neurons that represent X and Y coordinates of a person’s gaze on the screen. The CNN architecture for both eyes consists of 3 convolutional layers, 1 max pooling layer, 2 batch normalization layers, 3 dropout layers, 1 concatenate layer, 4 dense or fully-connected layers, and 1 final dense layer (the output layer).

**Figure 3:**
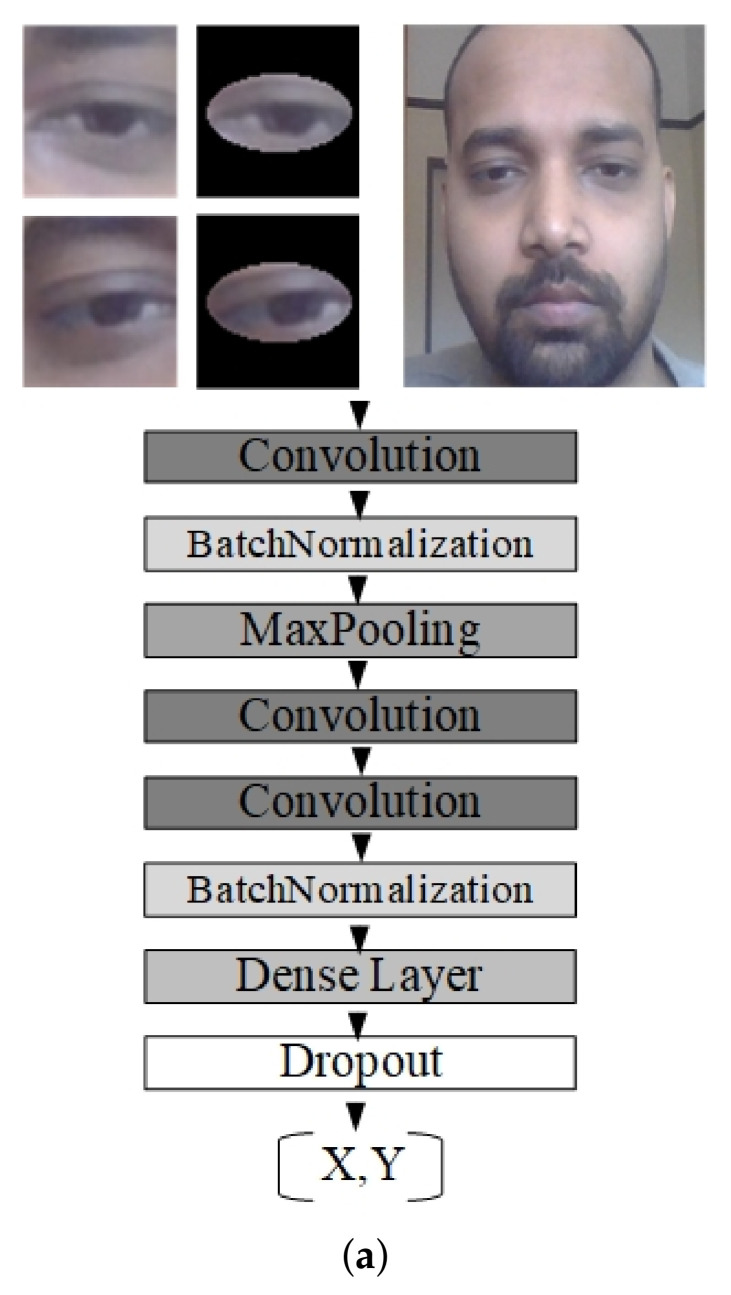

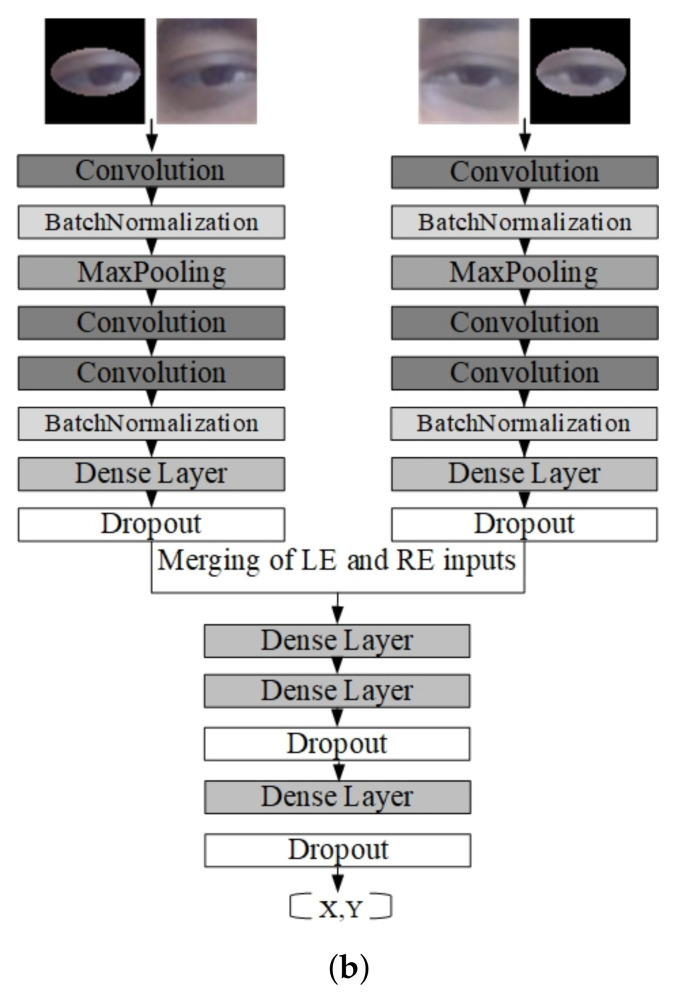
The architectures of the network used in the study. (**a**) Architecture that takes normal eyes, mask eyes, and face image as input. (**b**) Architecture that takes both left and right eyes (with or without mask) as a single input.

Figure 3 represents the architecture used in this study. Figure 3a shows the network architecture, which takes a single eye image (with or without mask) or face image as input. Figure 3b shows the network architecture that takes the left eye and right eye images as input. In Figure 3b, merging LE and RE represents a concatenation of outputs from left and right networks. The results from the concatenation layer go to dense layers and finally to the output layer.

### 3.4. Parameter Tuning

This section presents information about different parameters tuned while training the CNN network. We set different values for various parameters and searched for the best ones using the Keras tuner library [47]. We tracked the learning rate, dropout, and different kernel sizes for face and eyes with the fixed number of filters (kernels) in convolutional layers and the number of neurons in dense layers.

We checked the different dropout values and filter sizes for every learning rate for that experiment. Finally, we re-trained the model with the best values we achieved during the parameter tuning. Table 1 represents the list of the tuned parameters and their corresponding values. For instance, learning rate values were chosen from 0.1 up to 0.0001, and dropout was picked from 0.1 up to 0.5. Similarly, 3 × 3, 5 × 5, and 7 × 7 filter sizes were selected for the face experiment, and 3 × 3 and 5 × 5 filters were chosen for both eye experiments. We did not include a 7 × 7 kernel for eyes because eye image size was 64 × 64 pixels. We fixed the number of filters for the first layer to 32, for the second layer to 96, for the third layer to 160, and the number of dense units to 64. In Table 1, ConV1 represents the number of filters in the first layer, ConV2 for the second layer, and ConV3 for the third layer.

## 4. Results

In this section, we evaluate the performance of the CNN network on the person-specific dataset for gaze estimation. The models were trained using 80% of the data and tested using the remaining 20%. All experiments were performed on a moderate desktop computer with AMD Ryzen 7 3700X 8-core processor and 16 GB of RAM. Furthermore, all models were built using the TensorFlow library. The prediction quality of trained models was determined by calculating the MAE value in pixels.

The process of training a model on a local machine for eye and face on a desktop processor was time-consuming, taking an average of 12 h for the model using eye images and over 48 h for the model using face images. However, transferring the model to a machine with a graphics processing unit (GPU) for training resulted in a significant reduction in training time. The eye models were trained in an average of just 30 min, while the face models took only 1 h and 30 min on average. Looking at the time difference between training the model on the local machine and the machine with GPU, moving the model training to an external source was a good choice since it reduced training time to a great extent. To utilize the proposed method on a laptop or desktop, one needs to collect images in several sessions using the DataCollector application and train the model on them. The first time the model is trained on the images may take some time, but once trained, it can be used for gaze estimation.

The main goal of this experiment was to find the optimal model trained on low-quality images capable of predicting the person’s gaze. Two kinds of experiments were performed on the same dataset: one without masking the neighborhood of an eye and one with masking. Masking of the eye region was done using the OpenCV library. Every network was trained up to 100 epochs.

All results for both experiments are presented with different learning rates, dropout values, and filter sizes. Table 2, Table 3, Table 4 and Table 5 present errors in pixels for both left and right eyes without a mask (LE and RE) and with a mask (LEM and REM).

The first experiment compared the error for eyes with and without masking as input to the network to see if masking eye-neighboring pixels affects results. The best results are presented in Table 2, Table 3, Table 4 and Table 5 for different learning rates and filter sizes, with respect to both eyes with and without the mask. All the results are presented as MAE in pixels.

Similarly, the second experiment was intended to compare the results of the combination of both eyes with and without a mask. The best results for different learning rates and filter sizes are presented in Table 6. Finally, the results for the full face as a single input are shown in Table 7 with different learning rates and filter sizes.

We can observe from Table 2, Table 3, Table 4 and Table 5 that the network with learning rate 0.0001, filter size 5 × 5, and dropout rate 0.1 achieved the best results for both single-eye experiments (with and without mask). The best results are shown in bold. We found that the worst-performing network was the one with a learning rate of 0.1. This leads us to the conclusion that the learning rate is the most critical parameter affecting network performance, at least when considering this experiment.

The second experiment’s objective was to check if achieving results comparable to the single-eye input using two-eyed images as input in a more complex network was possible. We found a similar pattern as the single eye: a small learning rate is the most critical parameter following the filter size. However, the results were not as good as the single eye, and the training time was much longer than in single-eye experiments.

The final experiment was conducted with full-face images as input to the network for gaze prediction. The results for full-face images surprisingly showed very good results. We also included a 7 × 7 filter for the face image and got the best results with a 7 × 7 filter size, which suggests that a big filter size is a better choice than a small filter size (for example, 3 × 3) in this experiment.

Furthermore, we also experimented with different percentages of data in the training set to see how changing the amount of training data affects the accuracy. Table 8 presents the results of all experiments with a 3 × 3 filter size. We can observe that as we decreased the percentage of data in the training set, accuracy decreased as well. We used 80%, 60%, 40%, and 20% of images as the training data. As we can see in Table 8, the results with 20% data are the worst. This is because the number of training images (1880) is lower than the number of test images (2360). We also checked one more experiment with a lower dropout (0.005) to check if there would be any further improvement in accuracy. However, we found out that accuracy was not improving further.

**Figure 4:**
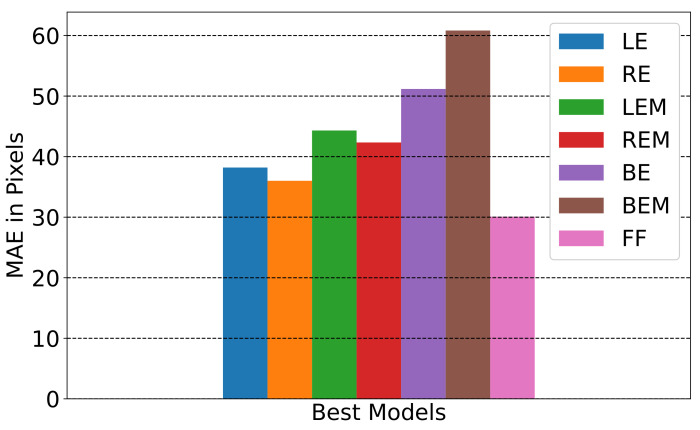
MAE in pixels for best models.

Since it is more common to present gaze errors in degrees than in pixels, we recalculated errors to give a better idea of our model’s performance. When a distance of a person from the camera and screen size is known, the error in pixels may be converted to degrees using the following formula:

(2) $$Error_{deg}=\tan^{-1}\frac{E_{cm}}{Dist},$$

where $E_{cm}$ is the error in cm after converting from pixels, and Dist is the distance between the screen and the person’s eyes in cm. The distance between the screen and the person varies, as we do not have any way to fix it without a chin rest. Therefore, we could only make an approximation of this distance. After that recalculation, the minimum error for LE is about 1.45 degrees. For the RE, the minimum error is about 1.37 degrees. For both eyes (BE), the minimum error is about 1.98 degrees. For full-face (FF), the minimum error is about 1.14 degrees. Figure 4 depicts the error of the best models in pixels.

## 5. Discussion

The experiments show that it is possible to achieve performance comparable to the performance of commercial eye trackers, which is, as stated in the introduction, from 0.5 to 2 degrees. The best result for RE images gave an error of 1.37 degrees and for FF images an error of 1.14 degrees, which is better than other similar webcam-based models from the literature. For example, in paper [40], the authors achieved 3.14 degrees of error on the MPIIGaze dataset; in paper [48], the authors achieved 3.94 degrees of error; and authors in [30] reported 4.8 degrees of error. Furthermore, in [20], the authors achieved 4.5 degrees of error on MPIIGaze and 10.3 degrees of error for the EYEDIAP dataset. Similarly, in paper [49], the authors achieved 2.8 degrees of error on MPIIGaze and 3.05 degrees of error on the EYEDIAP dataset. Paper [21] reported 3.34 degrees of error on the EYEDIAP dataset. Therefore, we can conclude that our results outperformed the previous research. However, it needs to be highlighted that the results were achieved for a person-specific model that uses the recordings of only one person and therefore are applicable only for this person’s eye/face images. Nevertheless, we have demonstrated that when the person-specific model is created using data from a standard webcam, it can achieve accuracy comparable to that of commercial eye trackers. Table 9 shows the comparison between our proposed method and other state-of-the-art methods. However, it is important to note that our method is trained on a single user, and other methods were trained on multiple people’s data. The comparison presented here is to show that if we train the model with a single user, we can achieve accuracy better than models trained on multiple people’s data.

The first experiment for the single eye with and without a mask (Table 2, Table 3, Table 4 and Table 5) shows that masking surrounding the area of the eyes did not help the network perform better compared to eyes without masking. The best result was for LE, which was 38.20 in MAE, achieved with a learning rate of 0.0001, filter size 5 × 5, and dropout 0.1. The best result for LEM was 44.32 with the same set of parameters. Similarly, the best value for RE was 36.01 and for REM was 42.34 with the same parameter settings. In all experiments for the single-eye input, we can see that the network for RE achieves the best results, 36.01, which shows that the network can learn good representations in middle layers that help the network achieve slightly better results than LE. However, we also observed some interesting results; for example, in Table 3 in one case, a 3 × 3 filter achieves a better result than 5 × 5 filter (LE at dropout 0.4) and REM achieved a better result than RE at 0.1, 0.2, and 0.3 dropout with a 5 × 5 filter size.

The combined input of both eyes achieved good results as well. In the case of a BE network with a learning rate of 0.001, filter size 5 × 5 and dropout 0.1 achieved the best result, 51.18 MAE, compared with a learning rate of 0.0001 and 3 × 3 filter size. However, the results were worse than for single images. This is perhaps due to the fact that, for both eyes, the network was harder to train, as it was more complex. With a 0.0001 learning rate, the network’s convergence was very slow, and because of that, the network was not able to reach its global minimum in 100 epochs. For BEM, the network achieved the best results with a learning rate of 0.0001, filter size 5 × 5, and dropout 0.1.

One of the most surprising findings among all experiments was the result of the model using full-face images (FF) as an input to the network. The CNN for FF achieved a value of 37.87 MAE, which is as good as single-eye input 36.01 (RE at 0.0001 learning rate, filter size 5 × 5, and dropout 0.1). Furthermore, it even achieved a better MAE score of 30.09 when the network was trained with a bigger filter size of 7 × 7. This interesting finding shows that using a bigger filter size gives a significant improvement. This is due to the fact that the face images were bigger, so a bigger filter size helped the network obtain more important features that ultimately helped the network achieve a better result. Similarly to others, the model with a learning rate of 0.1 was the worst performing, and it produced almost the same value for all filter sizes. Nevertheless, a good result for FF images suggests that it is possible to predict a person’s gaze even with a full-face image. The reason for the FF model’s good performance is probably the fact that the same face of the same person was used in both the training and testing phases.

## 6. Conclusions

In this study, we tried to answer the question of whether a person with limited hardware and software resources could reliably track gaze. This is an important issue because most commercial eye trackers are expensive, and not everyone can afford them. With the increasing number of eye-tracking applications in various fields, there is also a need to utilize low-cost eye tracking that can be used by any user without any specialized hardware or software. In this study, we trained a person-specific model that can be used on desktop or laptop devices without additional tools apart from a built-in camera. Our experimental findings show that the model created for one person gives much lower errors than the general models trained on multiple users’ data. We trained different CNNs with different parameter settings on images collected from the laptop webcam. In this study, we were able to show that, with properly tuned CNN architecture, it is possible to achieve good results for gaze estimation using a classic camera and that it can be useful in real-life applications.

The presented results are promising, yet there are many limitations correlated with this study. For example, we showed results for only one person, and we are still determining how well CNN will perform for other people since different people have different eye and facial appearances, and different image appearances affect network performance. We also performed all the experiments on a shallow CNN. In the future, we plan to check the results for complex networks with many layers (VGG, ResNet, Darknet, etc.) and check if the performance improves.

In our future research endeavors, we plan to address the abovementioned limitations. For this work, our motivation was to build a single model for a single person and see how well this model learns and works for a single user. For future work, we want to develop person-specific models for multiple users and check their performance. Furthermore, we also want to build one model for all users and compare it with the individual models. Moving forward, we also plan to include more subjects in data collection in order to study the generalized model for every subject as well as the specific model for every subject with the use of transfer learning.

## Figures and Tables

**Table 1:** List of networks’ parameters together with their values.

Parameter Name	Values
Learning Rate	(0.1, 0.01, 0.001, 0.0001)
Dropout	(0.1, 0.2, 0.3, 0.4, 0.5)
Kernel Size	(3 × 3), (5 × 5), (7 × 7)
ConV1 Filter No.	32
ConV2 Filter No.	96
ConV3 Filter No.	160
Dense Unit	64

**Table 2:** Results of LE and and RE with and without a mask (learning rate = 0.0001).

**Exp. Name**	LE	LE	LE	LE	LE	RE	RE	RE	RE	RE
**Filter Size**	3 × 3	3 × 3	3 × 3	3 × 3	3 × 3	3 × 3	3 × 3	3 × 3	3 × 3	3 × 3
**Dropout**	0.1	0.2	0.3	0.4	0.5	0.1	0.2	0.3	0.4	0.5
**Error**	**41.24**	42.55	44.92	50.30	55.37	**40.89**	42.97	46.83	48.39	47.37
**Exp. Name**	LE	LE	LE	LE	LE	RE	RE	RE	RE	RE
**Filter Size**	5 × 5	5 × 5	5 × 5	5 × 5	5 × 5	5 × 5	5 × 5	5 × 5	5 × 5	5 × 5
**Dropout**	0.1	0.2	0.3	0.4	0.5	0.1	0.2	0.3	0.4	0.5
**Error**	**38.20**	41.68	47.37	47.58	52.39	**36.01**	40.47	41.93	45.83	47.97
**Exp. Name**	LEM	LEM	LEM	LEM	LEM	REM	REM	REM	REM	REM
**Filter Size**	3 × 3	3 × 3	3 × 3	3 × 3	3 × 3	3 × 3	3 × 3	3 × 3	3 × 3	3 × 3
**Dropout**	0.1	0.2	0.3	0.4	0.5	0.1	0.2	0.3	0.4	0.5
**Error**	**50.26**	55.87	58.08	62.01	64.33	52.57	**51.86**	49.12	55.58	59.89
**Exp. Name**	LEM	LEM	LEM	LEM	LEM	REM	REM	REM	REM	REM
**Filter Size**	5 × 5	5 × 5	5 × 5	5 × 5	5 × 5	5 × 5	5 × 5	5 × 5	5 × 5	5 × 5
**Dropout**	0.1	0.2	0.3	0.4	0.5	0.1	0.2	0.3	0.4	0.5
**Error**	**44.32**	48.26	50.07	52.77	58.54	**42.34**	45.34	49.28	51.78	51.13

**Table 3:** Results of LE and and RE with and without a mask (learning rate = 0.001).

**Exp. Name**	LE	LE	LE	LE	LE	RE	RE	RE	RE	RE
**Filter Size**	3 × 3	3 × 3	3 × 3	3 × 3	3 × 3	3 × 3	3 × 3	3 × 3	3 × 3	3 × 3
**Dropout**	0.1	0.2	0.3	0.4	0.5	0.1	0.2	0.3	0.4	0.5
**Error**	**43.42**	45.04	50.09	49.35	53.40	**41.85**	44.15	47.63	47.27	54.64
**Exp. Name**	LE	LE	LE	LE	LE	RE	RE	RE	RE	RE
**Filter Size**	5 × 5	5 × 5	5 × 5	5 × 5	5 × 5	5 × 5	5 × 5	5 × 5	5 × 5	5 × 5
**Dropout**	0.1	0.2	0.3	0.4	0.5	0.1	0.2	0.3	0.4	0.5
**Error**	**42.10**	46.86	47.37	52.48	57.46	**37.74**	41.95	45.10	43.78	48.53
**Exp. Name**	LEM	LEM	LEM	LEM	LEM	REM	REM	REM	REM	REM
**Filter Size**	3 × 3	3 × 3	3 × 3	3 × 3	3 × 3	3 × 3	3 × 3	3 × 3	3 × 3	3 × 3
**Dropout**	0.1	0.2	0.3	0.4	0.5	0.1	0.2	0.3	0.4	0.5
**Error**	**55.53**	56.36	59.48	62.03	61.07	**52.58**	53.98	58.14	59.59	63.36
**Exp. Name**	LEM	LEM	LEM	LEM	LEM	REM	REM	REM	REM	REM
**Filter Size**	5 × 5	5 × 5	5 × 5	5 × 5	5 × 5	5 × 5	5 × 5	5 × 5	5 × 5	5 × 5
**Dropout**	0.1	0.2	0.3	0.4	0.5	0.1	0.2	0.3	0.4	0.5
**Error**	**48.93**	50.94	54.27	51.90	57.44	**43.95**	48.52	51.76	52.02	56.79

**Table 4:** Results of LE and RE with and without a mask (learning rate = 0.01).

**Exp. Name**	LE	LE	LE	LE	LE	RE	RE	RE	RE	RE
**Filter Size**	3 × 3	3 × 3	3 × 3	3 × 3	3 × 3	3 × 3	3 × 3	3 × 3	3 × 3	3 × 3
**Dropout**	0.1	0.2	0.3	0.4	0.5	0.1	0.2	0.3	0.4	0.5
**Error**	**47.13**	49.59	50.53	52.64	55.82	51.11	**51.03**	57.15	57.17	54.79
**Exp. Name**	LE	LE	LE	LE	LE	RE	RE	RE	RE	RE
**Filter Size**	5 × 5	5 × 5	5 × 5	5 × 5	5 × 5	5 × 5	5 × 5	5 × 5	5 × 5	5 × 5
**Dropout**	0.1	0.2	0.3	0.4	0.5	0.1	0.2	0.3	0.4	0.5
**Error**	**44.15**	50.69	49.53	54.63	61.59	**44.26**	48.73	51.54	50.94	58.63
**Exp. Name**	LEM	LEM	LEM	LEM	LEM	REM	REM	REM	REM	REM
**Filter Size**	3 × 3	3 × 3	3 × 3	3 × 3	3 × 3	3 × 3	3 × 3	3 × 3	3 × 3	3 × 3
**Dropout**	0.1	0.2	0.3	0.4	0.5	0.1	0.2	0.3	0.4	0.5
**Error**	**55.77**	60.04	65.93	69.06	66.52	**54.09**	66.11	59.81	61.64	68.88
**Exp. Name**	LEM	LEM	LEM	LEM	LEM	REM	REM	REM	REM	REM
**Filter Size**	5 × 5	5 × 5	5 × 5	5 × 5	5 × 5	5 × 5	5 × 5	5 × 5	5 × 5	5 × 5
**Dropout**	0.1	0.2	0.3	0.4	0.5	0.1	0.2	0.3	0.4	0.5
**Error**	**52.95**	67.42	72.61	63.52	66.90	**52.66**	59.46	74.77	67.51	69.32

**Table 5:** Results of LE and RE with and without a mask (learning rate = 0.1).

**Exp. Name**	LE	LE	LE	LE	LE	RE	RE	RE	RE	RE
**Filter Size**	3 × 3	3 × 3	3 × 3	3 × 3	3 × 3	3 × 3	3 × 3	3 × 3	3 × 3	3 × 3
**Dropout**	0.1	0.2	0.3	0.4	0.5	0.1	0.2	0.3	0.4	0.5
**Error**	**95.94**	280.29	272.36	354.21	279.89	**93.06**	389.72	308.64	184.71	250.27
**Exp. Name**	LE	LE	LE	LE	LE	RE	RE	RE	RE	RE
**Filter Size**	5 × 5	5 × 5	5 × 5	5 × 5	5 × 5	5 × 5	5 × 5	5 × 5	5 × 5	5 × 5
**Dropout**	0.1	0.2	0.3	0.4	0.5	0.1	0.2	0.3	0.4	0.5
**Error**	140.92	**131.15**	183.77	260.94	369.40	**111.65**	172.65	291.42	389.73	277.74
**Exp. Name**	LEM	LEM	LEM	LEM	LEM	REM	REM	REM	REM	REM
**Filter Size**	3 × 3	3 × 3	3 × 3	3 × 3	3 × 3	3 × 3	3 × 3	3 × 3	3 × 3	3 × 3
**Dropout**	0.1	0.2	0.3	0.4	0.5	0.1	0.2	0.3	0.4	0.5
**Error**	**216.23**	203.84	196.29	248.68	333.17	**104.11**	174.99	181.81	395.73	382.74
**Exp. Name**	LEM	LEM	LEM	LEM	LEM	REM	REM	REM	REM	REM
**Filter Size**	5 × 5	5 × 5	5 × 5	5 × 5	5 × 5	5 × 5	5 × 5	5 × 5	5 × 5	5 × 5
**Dropout**	0.1	0.2	0.3	0.4	0.5	0.1	0.2	0.3	0.4	0.5
**Error**	**96.16**	173.60	184.90	327.07	366.27	**150.37**	278.38	319.84	350.18	393.51

**Table 6:** Results of BE with and without a mask.

	Learning Rate = 0.0001					Learning Rate = 0.001				
**Exp. Name**	BE	BE	BE	BE	BE	BE	BE	BE	BE	BE
**Filter Size**	3 × 3	3 × 3	3 × 3	3 × 3	3 × 3	3 × 3	3 × 3	3 × 3	3 × 3	3 × 3
**Dropout**	0.1	0.2	0.3	0.4	0.5	0.1	0.2	0.3	0.4	0.5
**Error**	**53.08**	57.85	62.97	61.37	70.59	59.89	58.06	61.05	67.23	69.32
**Filter Size**	5 × 5	5 × 5	5 × 5	5 × 5	5 × 5	5 × 5	5 × 5	5 × 5	5 × 5	5 × 5
**Dropout**	0.1	0.2	0.3	0.4	0.5	0.1	0.2	0.3	0.4	0.5
**Error**	53.85	53.46	56.36	64.00	70.28	**51.18**	55.44	56.72	64.06	62.69
**Exp. Name**	BEM	BEM	BEM	BEM	BEM	BEM	BEM	BEM	BEM	BEM
**Filter Size**	3 × 3	3 × 3	3 × 3	3 × 3	3 × 3	3 × 3	3 × 3	3 × 3	3 × 3	3 × 3
**Dropout**	0.1	0.2	0.3	0.4	0.5	0.1	0.2	0.3	0.4	0.5
**Error**	66.39	66.02	73.64	73.45	85.3	64.19	71.90	76.04	78.35	82.71
**Filter Size**	5 × 5	5 × 5	5 × 5	5 × 5	5 × 5	5 × 5	5 × 5	5 × 5	5 × 5	5 × 5
**Dropout**	0.1	0.2	0.3	0.4	0.5	0.1	0.2	0.3	0.4	0.5
**Error**	**60.83**	62.75	63.90	69.83	73.56	**62.06**	64.87	66.76	70.06	71.80
	**Learning Rate = 0.01**					**Learning Rate = 0.1**				
**Exp. Name**	BE	BE	BE	BE	BE	BE	BE	BE	BE	BE
**Filter Size**	3 × 3	3 × 3	3 × 3	3 × 3	3 × 3	3 × 3	3 × 3	3 × 3	3 × 3	3 × 3
**Dropout**	0.1	0.2	0.3	0.4	0.5	0.1	0.2	0.3	0.4	0.5
**Error**	102.16	91.11	115.09	113.72	126.63	3680.40	368.50	373.74	368.68	368.62
**Filter Size**	5 × 5	5 × 5	5 × 5	5 × 5	5 × 5	5 × 5	5 × 5	5 × 5	5 × 5	5 × 5
**Dropout**	0.1	0.2	0.3	0.4	0.5	0.1	0.2	0.3	0.4	0.5
**Error**	**94.33**	106.30	125.27	102.14	121.40	370.49	370.51	370.49	372.40	**370.46**
**Exp. Name**	BEM	BEM	BEM	BEM	BEM	BEM	BEM	BEM	BEM	BEM
**Filter Size**	3 × 3	3 × 3	3 × 3	3 × 3	3 × 3	3 × 3	3 × 3	3 × 3	3 × 3	3 × 3
**Dropout**	0.1	0.2	0.3	0.4	0.5	0.1	0.2	0.3	0.4	0.5
**Error**	199.04	153.00	173.98	268.43	285.19	368.70	368.75	368.66	368.67	368.70
**Filter Size**	5 × 5	5 × 5	5 × 5	5 × 5	5 × 5	5 × 5	5 × 5	5 × 5	5 × 5	5 × 5
**Dropout**	0.1	0.2	0.3	0.4	0.5	0.1	0.2	0.3	0.4	0.5
**Error**	**127.14**	180.67	175.08	233.90	337.41	366.14	**366.10**	368.10	**366.10**	366.90

**Table 7:** Results of full-face.

	Learning Rate = 0.0001					Learning Rate = 0.001				
**Exp. Name**	FF	FF	FF	FF	FF	FF	FF	FF	FF	FF
**Filter Size**	3 × 3	3 × 3	3 × 3	3 × 3	3 × 3	3 × 3	3 × 3	3 × 3	3 × 3	3 × 3
**Dropout**	0.1	0.2	0.3	0.4	0.5	0.1	0.2	0.3	0.4	0.5
**Error**	39.38	42.29	43.26	47.76	64.33	40.24	45.49	54.17	54.42	64.33
**Filter Size**	5 × 5	5 × 5	5 × 5	5 × 5	5 × 5	5 × 5	5 × 5	5 × 5	5 × 5	5 × 5
**Dropout**	0.1	0.2	0.3	0.4	0.5	0.1	0.2	0.3	0.4	0.5
**Error**	37.87	43.04	39.04	49.59	59.02	**37.75**	55.84	62.60	61.84	77.63
**Filter Size**	7 × 7	7 × 7	7 × 7	7 × 7	7 × 7	7 × 7	7 × 7	7 × 7	7 × 7	7 × 7
**Dropout**	0.1	0.2	0.3	0.4	0.5	0.1	0.2	0.3	0.4	0.5
**Error**	**30.09**	34.12	43.60	47.17	52.73	55.52	60.83	54.71	54.03	59.80
	**Learning Rate = 0.01**					**Learning Rate = 0.1**				
**Exp. Name**	FF	FF	FF	FF	FF	FF	FF	FF	FF	FF
**Filter Size**	3 × 3	3 × 3	3 × 3	3 × 3	3 × 3	3 × 3	3 × 3	3 × 3	3 × 3	3 × 3
**Dropout**	0.1	0.2	0.3	0.4	0.5	0.1	0.2	0.3	0.4	0.5
**Error**	47.48	76.70	273.03	81.23	90.77	**380.65**	380.65	380.64	380.65	380.65
**Filter Size**	5 × 5	5 × 5	5 × 5	5 × 5	5 × 5	5 × 5	5 × 5	5 × 5	5 × 5	5 × 5
**Dropout**	0.1	0.2	0.3	0.4	0.5	0.1	0.2	0.3	0.4	0.5
**Error**	**43.24**	97.59	169.95	57.20	80.20	380.65	380.65	380.65	380.65	380.65
**Filter Size**	7 × 7	7 × 7	7 × 7	7 × 7	7 × 7	7 × 7	7 × 7	7 × 7	7 × 7	7 × 7
**Dropout**	0.1	0.2	0.3	0.4	0.5	0.1	0.2	0.3	0.4	0.5
**Error**	53.19	77.66	78.16	64.05	82.59	380.65	380.65	380.65	380.65	380.65

**Table 8:** Results of different training sets on face data.

	Learning Rate = 0.0001					Learning Rate = 0.001				
**Exp. Name**	FF(80)	FF(80)	FF(80)	FF(80)	FF(80)	FF(80)	FF(80)	FF(80)	FF(80)	FF(80)
**Filter Size**	3 × 3	3 × 3	3 × 3	3 × 3	3 × 3	3 × 3	3 × 3	3 × 3	3 × 3	3 × 3
**Dropout**	0.1	0.2	0.3	0.4	0.5	0.1	0.2	0.3	0.4	0.5
**Error**	41.88	**41.87**	43.81	47.89	60.35	**45.90**	52.25	52.17	59.46	75.87
**Exp. Name**	FF(60)	FF(60)	FF(60)	FF(60)	FF(60)	FF(60)	FF(60)	FF(60)	FF(60)	FF(60)
**Dropout**	0.1	0.2	0.3	0.4	0.5	0.1	0.2	0.3	0.4	0.5
**Error**	**42.88**	44.94	49.04	53.55	60.35	**47.33**	54.69	55.17	60.23	74.87
**Exp. Name**	FF(40)	FF(40)	FF(40)	FF(40)	FF(40)	FF(40)	FF(40)	FF(40)	FF(40)	FF(40)
**Dropout**	0.1	0.2	0.3	0.4	0.5	0.1	0.2	0.3	0.4	0.5
**Error**	49.37	**48.70**	54.30	52.43	58.83	**50.60**	59.03	63.97	57.37	70.62
**Exp. Name**	FF(20)	FF(20)	FF(20)	FF(20)	FF(20)	FF(20)	FF(20)	FF(20)	FF(20)	FF(20)
**Dropout**	0.1	0.2	0.3	0.4	0.5	0.1	0.2	0.3	0.4	0.5
**Error**	**56.17**	59.29	67.03	68.24	71.01	63.36	**61.89**	76.18	74.96	71.11
	**Learning Rate = 0.01**					**Learning Rate = 0.1**				
**Exp. Name**	FF(80)	FF(80)	FF(80)	FF(80)	FF(80)	FF(80)	FF(80)	FF(80)	FF(80)	FF(80)
**Filter Size**	3 × 3	3 × 3	3 × 3	3 × 3	3 × 3	3 × 3	3 × 3	3 × 3	3 × 3	3 × 3
**Dropout**	0.1	0.2	0.3	0.4	0.5	0.1	0.2	0.3	0.4	0.5
**Error**	**58.01**	81.48	71.18	125.93	150.27	**377.40**	377.40	377.41	377.40	377.41
**Exp. Name**	FF(60)	FF(60)	FF(60)	FF(60)	FF(60)	FF(60)	FF(60)	FF(60)	FF(60)	FF(60)
**Dropout**	0.1	0.2	0.3	0.4	0.5	0.1	0.2	0.3	0.4	0.5
**Error**	**59.21**	83.88	70.45	127.45	148.76	377.41	**377.40**	377.41	377.40	377.41
**Exp. Name**	FF(40)	FF(40)	FF(40)	FF(40)	FF(40)	FF(40)	FF(40)	FF(40)	FF(40)	FF(40)
**Dropout**	0.1	0.2	0.3	0.4	0.5	0.1	0.2	0.3	0.4	0.5
**Error**	**53.98**	114.24	125.67	82.95	136.86	382.99	**382.99**	382.99	382.99	382.99
**Exp. Name**	FF(20)	FF(20)	FF(20)	FF(20)	FF(20)	FF(20)	FF(20)	FF(20)	FF(20)	FF(20)
**Dropout**	0.1	0.2	0.3	0.4	0.5	0.1	0.2	0.3	0.4	0.5
**Error**	**61.68**	112.39	263.82	139.86	389.31	**465.23**	465.34	464.79	465.34	465.34

**Table 9:** Angular errors of our method in comparison with other state-of-the-art methods.

Method	Error in Degrees
FAZE [40]	3.14
L2CS-Net [48]	3.94
RT-Gene [30]	4.8
Deep Pictorial Gaze [20]	4.5
Multi-stream CNN [49]	2.8
Recurrent CNN [21]	3.34
Ours (with RE)	**1.37**
Ours (with FF)	**1.14**

## Data Availability

The dataset used in the research is available at https://github.com/faizygithub/PersonSpecificGazeEstimation (accessed on 8 April 2023).

## Abbreviations

The following abbreviations are used in this manuscript:

CNN	Convolutional Neural Networks
GPU	Graphics Processing Unit
VOG	Video Oculography
VJ	Viola–Jones
LE	Left Eye
RE	Right Eye
LEM	Left Eye Masked
REM	Right Eye Masked
BE	Both Eyes
BEM	Both Eyes Masked
FF	Full Face
